# Pharmacokinetic Variability and Target Attainment of Fluconazole in Critically Ill Patients

**DOI:** 10.3390/microorganisms9102068

**Published:** 2021-09-30

**Authors:** Ruth Van Daele, Joost Wauters, Katrien Lagrou, Raphaël Denooz, Marie-Pierre Hayette, Matthias Gijsen, Roger J. Brüggemann, Yves Debaveye, Isabel Spriet

**Affiliations:** 1 Clinical Pharmacology and Pharmacotherapy, Department of Pharmaceutical and Pharmacological Sciences, KU Leuven, 3000 Leuven, Belgium; matthias.gijsen@uzleuven.be (M.G.); isabel.spriet@uzleuven.be (I.S.); 2 Pharmacy Department, University Hospitals Leuven, 3000 Leuven, Belgium; 3 Laboratory for Clinical Infectious and Inflammatory Disorders, Department of Microbiology, Immunology and Transplantation, KU Leuven, 3000 Leuven, Belgium; joost.wauters@uzleuven.be; 4 Medical Intensive Care Unit, University Hospitals Leuven, 3000 Leuven, Belgium; 5 Clinical Department of Laboratory Medicine and National Reference Centre for Mycosis, Excellence Centre for Medical Mycology (ECMM), University Hospitals Leuven, 3000 Leuven, Belgium; katrien.lagrou@uzleuven.be; 6 Department of Microbiology, Immunology and Transplantation, KU Leuven, 3000 Leuven, Belgium; 7 Laboratory of Clinical, Forensic, Environmental and Industrial Toxicology, CHU Sart-Tilman, University of Liège, 4000 Liège, Belgium; raphael.denooz@chuliege.be; 8 Laboratory of Clinical Microbiology, Centre for Interdisciplinary Research on Medicines (CIRM), University of Liège, 4000 Liège, Belgium; mphayette@chuliege.be; 9 Department of Pharmacy and Radboud Institute for Health Sciences, Radboud University Medical Center, 6525 GA Nijmegen, The Netherlands; roger.bruggemann@radboudumc.nl; 10 Center of Expertise in Mycology Radboudumc/CWZ, Radboud University Medical Center, 6525 GA Nijmegen, The Netherlands; 11 Laboratory of Intensive Care Medicine, Department of Cellular and Molecular Medicine, KU Leuven, 3000 Leuven, Belgium; yves.debaveye@uzleuven.be; 12 Intensive Care Unit, University Hospitals Leuven, 3000 Leuven, Belgium

**Keywords:** fluconazole, critically ill patients, pharmacokinetics, target attainment, variability, exposure

## Abstract

*Background*: Fluconazole is one of the oldest antifungal drugs. Previous studies have raised concerns considering variability in exposure and inadequate target attainment in critically ill patients. The current study aims to define variability and target attainment for fluconazole exposure in a large group of critically ill patients. *Methods*: In this pharmacokinetic study, daily plasma trough samples and, if possible, 24 h urine samples were collected to determine fluconazole concentration. A minimum target trough concentration of 10–15 mg/L was selected, corresponding to a free area under the concentration–time curve above the minimum inhibitory concentration (*f*AUC/MIC) of at least 100 for an MIC of 4 mg/L. Covariates that significantly influenced fluconazole exposure were identified. *Results*: In total, 288 plasma samples from 43 patients, with a median age of 66 years, were included. The median fluconazole trough concentration was 22.9 mg/L. A notable component of the measured concentrations was below the target trough concentrations (13% <10 mg/L and 27% <15 mg/L). The intra- and intersubject variability were 28.3% and 50.5%, respectively. The main covariates determining fluconazole exposure were the administered dose (mg/kg), augmented renal clearance, and renal replacement therapy. *Conclusions*: Fluconazole trough concentrations are variable in critically ill patients and a considerable number of these concentrations was below the predefined target trough concentrations.

## 1. Introduction

Fluconazole is one of the oldest antifungal drugs and is used for the treatment of invasive candidiasis and candidaemia. Despite the extensive experience with this drug in clinical practice, also at intensive care units (ICUs), few data are available concerning exposure and target attainment (TA). Since fluconazole is predominantly renally excreted as an unchanged drug (80%) via glomerular filtration, followed by tubular reabsorption, alterations in renal function can influence fluconazole exposure [1]. Renal function can be expressed based on measured 24 h urinary creatinine clearance (CrCL_24h_) or via formulae that estimate the glomerular filtration rate (eGFR). Despite the fact that CrCL_24h_ is a better estimator of renal function, it is not always routinely performed [2]. Moreover, critical illness might influence fluconazole exposure due to pathophysiological changes such as altered absorption, increased volume of distribution (Vd), hypoalbuminemia, influenced hepatic metabolism, or renal elimination and the use of extracorporeal circuits [3].

The scarce available fluconazole exposure data frequently focus on exposure in obese patients and those undergoing continuous renal replacement therapy (CRRT) [4,5,6,7]. In critically ill patients, a subanalysis of the DALI study reported fluconazole concentrations over one dosing interval (*n* = 15) and revealed a large interindividual variability with underexposure to fluconazole in 5 out of the 15 patients, likely due to underdosing (<6 mg/kg) [8]. Another pharmacokinetic (PK) study (*n* = 19) confirmed highly variable fluconazole clearance and the need for higher-than-standard doses, i.e., >400 mg once daily, in critically ill patients in order to attain the predefined exposure targets—especially in patients with an adequate to augmented renal clearance or CRRT [9]. A recent PK study (*n* = 49) reported substantial variability in fluconazole plasma concentrations in critically ill patients, with underexposure detected in the majority of them [10].

Invasive candidiasis and candidaemia are associated with high morbidity and mortality [11,12]. The European Society of Clinical Microbiology and Infectious Diseases (ESCMID) guideline for diagnosis and management of *Candida* disease recommends the use of echinocandins as first-line targeted therapy for these infections [13]. Especially in critically ill patients, echinocandins are preferred to fluconazole [14] as a post hoc analysis of the Reboli trial indicated that anidulafungin was more effective than fluconazole in the treatment of severely ill patients [15]. However, the latter trial was designed to only document noninferiority and, in addition, a more recent study revealed no trend toward lower efficacy of fluconazole, as compared with echinocandins, even in patients with severe sepsis or shock [16]. Advantages of fluconazole include its acceptable safety profile, minimal hepatic metabolism, extensive distribution into body fluids and tissues, low protein binding (11%–12%), and low cost [1,17,18,19].

Fluconazole plasma concentrations are not monitored in routine clinical practice, but pharmacokinetic and pharmacodynamic (PKPD) targets have been defined. The European Committee on Antimicrobial Susceptibility Testing (EUCAST) recommends a free area under the concentration–time curve above the minimum inhibitory concentration (*f*AUC/MIC) of at least 100 [20]. Studies have shown that AUC correlates well with plasma trough concentration, which is more practical to use in clinical settings [10,21]. In cases of *Candida* spp. with a MIC value of up to 4 mg/L, a *f*AUC of 400 is targeted, corresponding to trough concentrations of around 10–15 mg/L [10,22].

Since underexposure to fluconazole in critically ill patients has been suggested in the literature, our study aimed to longitudinally assess the target attainment and variability of fluconazole exposure, based on plasma trough concentrations, in a large group of critically ill patients.

## 2. Materials and Methods

### 2.1. Study Design and Population

All adult, critically ill patients treated with fluconazole between 30 April 2019 and 5 March 2020 in the University Hospitals Leuven were eligible for inclusion in this prospective, observational PK study, irrespective of timing of fluconazole initiation (before or during ICU stay), provided that there were no therapeutic restrictions and written informed consent was obtained from the patient or their relatives. Fluconazole indication, dose, and route of administration were at the discretion of the treating physician. No formal sample size calculation was performed since this was an exploratory study. The present study was conducted in accordance with the Declaration of Helsinki and good clinical practice regulation, and was approved by the Ethics Committee Research UZ/KU Leuven (S62242, 20 February 2019). This study was registered at ClinicalTrials.gov (NCT04252027). The primary objective was to longitudinally document fluconazole trough concentrations (C_min_) and to determine TA based on these concentrations. As secondary objectives, this study aimed to determine intra- and intersubject variability on C_min_ and covariates that might explain this variability.

### 2.2. Sample Collection

One daily plasma trough sample was collected over a maximum of 15 consecutive days. Fewer sampling days were possible, e.g., in cases where the patient was transferred to another (non-ICU) ward or when fluconazole treatment was ceased. Only trough samples were included in our analysis, defined as samples that were collected 24 h (±1 h) or 12 h (±1 h) after the previous administered dose for a once daily or twice daily dosing regimen, respectively. Blood samples were collected in lithium-heparin-containing tubes. The samples were centrifuged for ~10 min at 1000× *g* and stored at −80 °C until analysis. In a subset of patients, daily urine samples from 24 h urine collection periods were also collected and stored at −80 °C until analysis.

### 2.3. Method of Analysis

The analytical method used for fluconazole quantification was previously validated and published [23]. In brief, liquid–liquid extraction was used for sample preparation before samples were analyzed via an ultra-performance liquid chromatography method with diode-array detection. This assay was linear from 0.3 to 10 mg/L for fluconazole with bias and imprecision values for intra- and interassays lower than 10% and 15%, respectively. This analytical method was used for fluconazole analysis of both plasma and urine but was only previously validated for plasma. Nonetheless, the primary goal of the fluconazole quantification in urine was not to determine whether or not a certain target concentration was attained, but if a correlation with the plasma concentration was observed. Urine was sampled after a 24 h urine collection period and stability for seven days at room temperature has been demonstrated (data unpublished).

### 2.4. Data Collection 

The following parameters describing the study population were collected: age, sex, body weight, BMI, and length of ICU stay. Covariates that may influence fluconazole exposure (see Introduction) were also collected: the administered dose (mg/kg), the day of fluconazole administration, the attainment of steady state (≥day 2 if loading dose was administered and ≥day 5 if no loading dose was administered or after a change in dose [1]), the mode of administration, serum creatinine, augmented renal clearance (ARC), and CRRT (continuous venovenous hemofiltration (CVVH) and intermittent hemodialysis (IHD)). To account for the possible influence of critical illness, the Acute Physiology and Chronic Health Evaluation II (APACHE II) score (calculated using the Apache II calculator of ClinCalc [24]), the Sequential Organ Failure Assessment (SOFA) score, treatment with ECMO, total bilirubin, alkaline phosphatase, and hematocrit were also collected. Values under or above the limit of detection were replaced by the minimum or maximum measurable value, respectively.

### 2.5. Variability and Target Attainment

Intersubject and intrasubject coefficients of variation (%CV) were calculated using a linear mixed model with a random intercept by dividing the estimated standard deviation (of the random effects’ intercept and residuals, respectively) by the fixed effects’ estimate of the intercept. The lower limit for fluconazole trough concentration was defined as 10–15 mg/L, as recommended by the ECIL-6 guidelines [22].

### 2.6. Statistical Analysis

For descriptive statistics, continuous variables were expressed as median (interquartile range (IQR)) and categorical variables as ratio (percentage). To identify covariates potentially impacting fluconazole exposure, generalized estimating equation (GEE) logistic regression with backward covariate selection was applied using fluconazole trough concentration as a continuous outcome variable. Thirteen covariates (fluconazole dose, day of fluconazole administration, attainment of steady state, mode of administration, serum creatinine, ARC, CVVH, IHD, SOFA score, ECMO, total bilirubin, alkaline phosphatase, and hematocrit) were tested in the multivariate analysis, based on their presumed influence on fluconazole exposure. Two different multivariate analyses were performed, based on two different definitions of ARC—namely, CrCL**_24h-1_** above 130 mL/min/1.73 m^2^ and eGFR above 96.5 mL/min/1.73 m^2^ (CKD-Epi) [2]—since CrCL**_24h_** data were missing in a large subset of the included patients. For statistical analysis, missing continuous data were completed with the median value for the same patient, if available, or the median of the total population. The significance level was set to 0.05. All statistical analyses were performed using R software (R version 3.6.3; The R Foundation for Statistical Computing, Vienna, Austria).

## 3. Results

### 3.1. Study Population

Plasma samples were collected in 43 patients with a median (interquartile range (IQR)) age, body weight, and APACHE II score of 66 (58–70) years, 70 (61–87) kg, and 18 (14–24), respectively (Table 1). Renal function and renal replacement therapy are presented in Table 2. In total, 288 fluconazole trough concentrations were included in this analysis, with a median (IQR) trough concentration of 22.9 (14.7–35.2) mg/L. Fifty-one urine samples from 19 patients were analyzed.

### 3.2. Variability and Target Attainment

**Figure 1:**
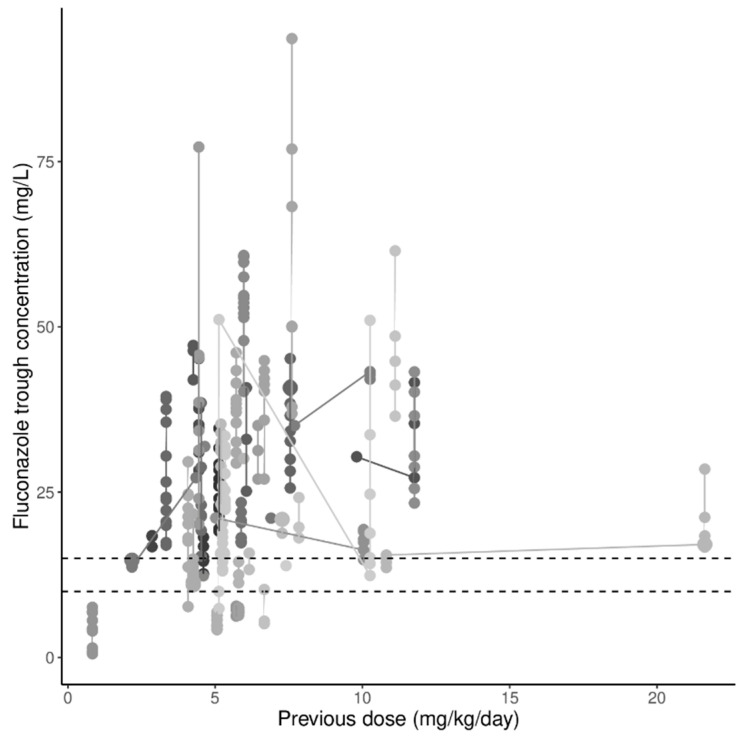
Fluconazole trough concentrations as a function of the previously administered daily dose. The grey scale represents concentrations in individual patients. Points of individual patients are connected with a solid line. The horizontal dashed lines depict the 10 and 15 mg/L thresholds.

**Figure 2:**
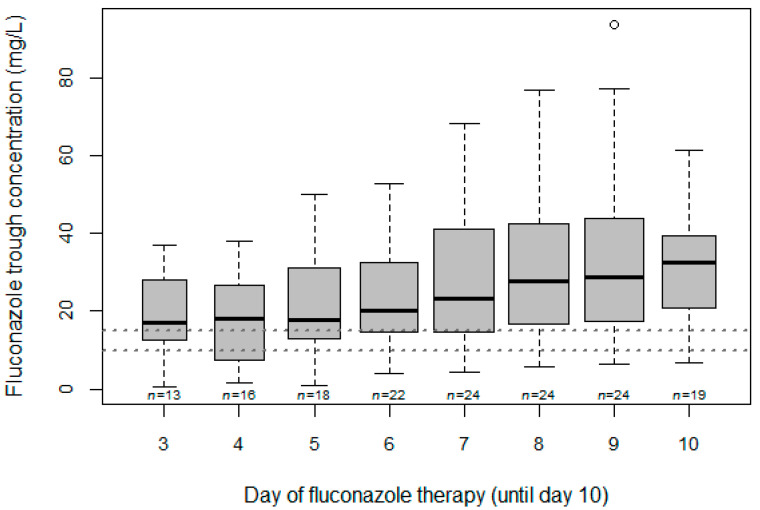
Boxplot of fluconazole trough concentrations during the first 10 days of fluconazole therapy (*n* = 160). The boxes represent the median and IQR. The horizontal lines depict the 10 and 15 mg/L thresholds.

In Figure 1, fluconazole trough concentrations are depicted as a function of the administered dose. A notable component of the measured concentrations was below the lower target concentrations (13% <10 mg/L and 27% <15 mg/L). In Figure 2, fluconazole concentrations are presented as a function of the day of fluconazole treatment (until day 10, since there were considerably less samples collected >10 days after treatment). The intra- and intersubject variability (%CV) were 28.3% and 50.5%, respectively.

### 3.3. Multivariate Analysis

In Table 3, covariates significantly influencing fluconazole exposure are reported together with their *p*- and beta-values. Covariates which significantly increased fluconazole trough exposure included the administered dose (mg/kg) and—in multivariate analysis 2 alone—oral administration and elevated alkaline phosphatase concentration. The presence of ARC and CVVH significantly decreased fluconazole trough concentrations.

### 3.4. Correlation Plasma and Urine Samples

**Figure 3:**
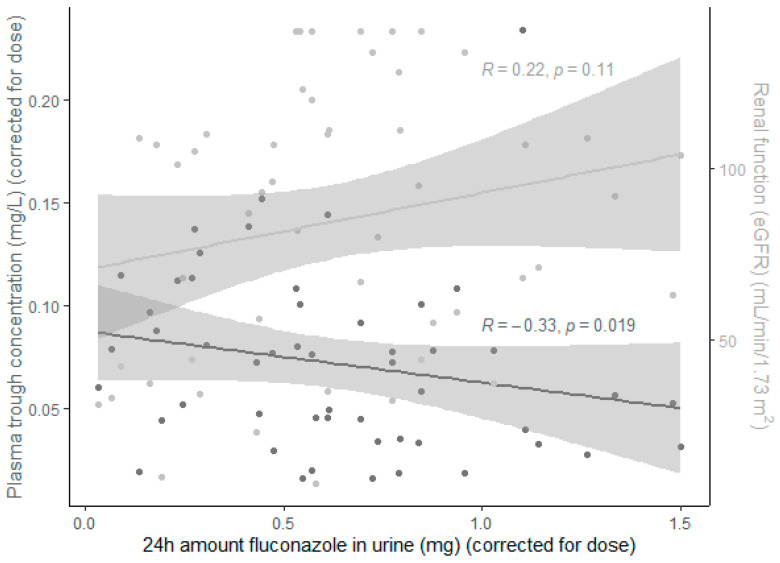
Correlation between fluconazole and renal function. Regression line, Spearman correlation coefficient, and *p*-value of the correlation between the amount of fluconazole (mg) found in the urine following a 24 h collection period (corrected for the administered dose) and both the fluconazole plasma trough concentration (corrected for the administered dose, dark grey) and the renal function (eGFR, light grey).

As seen in Figure 3, the amount of fluconazole (mg) detected in urine following a 24 h collection period (corrected for the administered dose) correlated negatively with the fluconazole plasma trough concentration (corrected for the administered dose) and positively with the renal function (eGFR).

## 4. Discussion

In this study, target attainment for fluconazole trough concentrations was determined in a large cohort of critically ill patients. The median (IQR) fluconazole trough concentration was 22.9 (14.7–35.2) mg/L. In 13% to 27% of the samples, the lower limit for efficacy was not attained for *Candida* spp with an MIC-value of 4 mg/L. Intersubject variability and underexposure were observed, likely due to low administered doses (mg/kg), ARC, and CRRT.

Few data are available on fluconazole exposure, especially concerning trough concentrations. In one study, trough samples from 28 noncritically ill hematology patients receiving daily doses of 200 mg oral fluconazole for at least one week revealed a median predose serum concentration of 5.6 m/L with a range of 0.11–18 mg/L [25]. In another study of 11 bone marrow transplant patients receiving 200 mg (or 100 mg in one patient) of fluconazole orally once daily, the mean fluconazole C_min_ were dependent on the duration of administration (D1: 1.59 mg/L; D13: 4.35 mg/L; and D27: 7.96 mg/L) [26]. Based on visual inspection of Figure 2 and Table 4, exposure and target attainment seem to be time-dependent in our study over the first 10 days of fluconazole treatment, but the day of treatment did not significantly impact C_min_ in the multivariate analyses. Subtherapeutic trough concentrations were indeed observed during the entire treatment period (23.4% on days ≤5, 32.5% between days 5–10, and 44.1% on days >10). A loading dose seems important to achieve early adequate exposure, as illustrated by the difference in median C_min_ during the first 5 days of fluconazole treatment for patients with (*n* = 18, 19.4 mg/L) versus without (*n* = 5, 13.3 mg/L) a loading dose. However, the attainment of steady state was not withheld in the final model of our multivariate analysis. Two studies reported fluconazole C_min_ in critically ill patients. In the DALI study, administering a median [IQR] dose of 400 (200–400) mg or 4.9 (2.3–5.0) mg/kg resulted in a mean fluconazole C_min_ of 14 mg/L [8]. Boonstra et al. reported C_min_ values of 6.4 mg/L and 13.3 mg/L on day 1 and 5 of treatment, respectively, in 21 critically ill patients treated with 400 mg fluconazole 24 h after a loading dose of 800 mg [10]. Our median trough concentration was higher than that reported in all of the above studies. This may be partially explained by the higher administered doses, also in patients undergoing CVVH (Table 2).

A moderate intrasubject (28.3%) and considerable intersubject (50.5%) variability in fluconazole plasma exposure were observed with the administered dose (mg/kg), CVVH and ARC being important determinants for fluconazole exposure. The importance of the fluconazole dose was reported in the DALI study, in which a maintenance dose of 6 mg/kg instead of the fixed 400 mg dose was advocated [8]. Moreover, our study confirmed that renal function is an important driver of fluconazole exposure, which was also observed in earlier reports [10]. A daily dose of 600 mg for patients with adequate renal function, and 800 mg for patients undergoing CRRT, was previously recommended based on a pharmacokinetic evaluation of 19 patients [9]. A dose of 800 mg was previously suggested for patients treated with CVVH, based on exposure in eight patients [27]. The need for dose adjustment based on renal function can be explained by the predominant renal excretion of unchanged fluconazole and the reduction, or even absence, of tubular reabsorption in the case of CRRT [1,6,27]. With this in mind, we hypothesized that plasma urea concentrations might also correlate with fluconazole exposure, since renal handling of urea also comprises glomerular filtration and tubular reabsorption [17,28]. Plasma urea concentration was taken into account—instead of serum creatinine—in the first multivariate analysis (as a separate analysis, results not shown), and was retained as a significant covariate (*p* = 0.016, beta = 0.10).

The presence of ARC, regardless of the definition, had an important impact on fluconazole exposure (Table 3). This is an important finding since most published research focuses on patients with impaired renal function and the associated need for RRT [5,6,7]. ARC is of significant clinical importance in critically ill patients, with reported a prevalence of between 16% and 100%. Additionally, when present, ARC persists for several days [29]. ARC is more prevalent in (cardiac) surgery patients, which is also a risk factor for the development of candidiasis, as illustrated by the Candida score [29,30]. Since the excretion of fluconazole increases with augmented renal clearance (Figure 3), future efforts should, in our opinion, focus on modeling fluconazole exposure in patients with ARC so that optimized dosing regimens can be proposed for this important subgroup of critically ill patients.

Only three patients (28 C_min_) received oral fluconazole. Surprisingly, in the second multivariate analysis, higher fluconazole concentrations were linked to oral administration, with relatively high beta-values. However, in the univariate analysis, an inverse relationship was observed. This is probably due to confounding factors in the multivariate analysis, which can presumably be explained by the severity of illness, with less severely ill patients having a higher chance of receiving oral administration.

In the second multivariate analysis, elevated alkaline phosphatase was associated with higher fluconazole exposure but with very weak impact (beta = 0.04). Only 11% of the administered fluconazole dose is excreted as (hepatic) metabolites [31]; therefore, it is unlikely that an elevated alkaline phosphatase concentration leads to higher fluconazole exposure. By contrast, increased alkaline phosphatase is mostly considered to be an indicator of cholestasis, which might be caused by higher fluconazole exposure [32].

This study has some limitations. First, the main goal of our study was to longitudinally asses fluconazole exposure, so only trough concentrations were collected. As a result, we were not able to perform population PK modeling on our data. Moreover, the PKPD target was defined based on *f*AUC/MIC values, so the collection of multiple plasma samples during one dosing interval may have yielded interesting data. However, the collection of trough samples is most achievable in clinical practice and a correlation between AUC and C_min_ has previously been demonstrated and targets based on C_min_ have been defined. Second, we did not consider an upper limit for exposure since no clear upper limit has been defined; however, it is known that fluconazole has a broad therapeutic index [22]. Fluconazole-induced convulsions are reported at trough concentrations of approximately 80 mg/L, and only one of our measured concentrations exceeded this value [33]. Finally, this was an exploratory, observational study and as such no clinical endpoints were evaluated.

Important in the interpretation of our results is the fact that the target *f*AUC and associated C_min_ were defined based on an MIC value of 4 mg/L, which is the *Candida* nonspecies-related EUCAST breakpoint for resistance [20]. In the specific case of *C. glabrata*, which demonstrates intermediate susceptibility up to MIC values of 16 mg/L, higher doses of fluconazole are mandatory [20]. However, *Candida* spp. other than *C. glabrata* are often associated with MIC values lower than 4 mg/L. Based on the EUCAST MIC distribution for *C. albicans, C. dubliniensis, C. parapsilosis, C. tropicalis*, and *C. kefyr*, the ranges of MIC50 and MIC90 values are 0.25–0.5 mg/L and 0.5–2 mg/L, respectively [20]. A European multicenter study found MIC50 and MIC90 values ranging from 0.12–0.5 mg/L and 0.25–4 mg/L for susceptible *C. albicans*, respectively [34]. A Belgian study reported fluconazole MIC50 and MIC90 values of 0.25 and 2 mg/L for *C. albicans*, respectively [35]. Therefore, non-glabrata *Candida* spp. with an MIC value of 4 mg/L occur but are rather rare, hence using this cut-off in the calculation of TA might be too strict, depending on geographic location. In most European settings, an MIC value of 2 mg/L might be a better estimate for most non-glabrata *Candida* spp. Consequently, when considering a two-fold lower MIC value, a trough concentration of 7.5 mg/L, instead of 15 mg/L, could be targeted. In that case, the TA in our study population would be 89%, which is considerably better than 73% and leaves only a discrete margin for improvement. The 11% samples without TA could be explained by ARC (*n* = 17, 52%), CVVH (*n* = 10, 30%), administered dose (*n* = 1, 3%), or the one patient (five C_min_) with both oral administration and a high (but not augmented) renal clearance (93–96 mL/min/1.73 m^2^) (*n* = 5, 15%).

Different strategies to guide fluconazole dosage in critically ill patients exist. Boonstra et al. [10] underlined the role of individualized dosing based on therapeutic drug monitoring (TDM) and predicted an increased risk of adverse events with routine administration of higher fluconazole dosages. Sandaradura et al. [36] compared three different dosing strategies in a virtual ICU population: standard guideline dosing, TDM-guided dosing, and model-optimized dosing based on patient characteristics such as weight, gender, age, serum creatinine, and CRRT status. Considerable underexposure was observed in the first two strategies (TA, based on an MIC value of 2 mg/L, was between 63% and 79%) while the model-optimized dosing strategy resulted in ≥ 98% TA. In both of these studies, the extensive variability of fluconazole exposure in critically ill patients was acknowledged, which is in accordance with our conclusions. However, although TDM and model-optimized dosing might be reasonable options to optimize TA, these strategies are associated with some disadvantages. In the case of TDM, delay time and additional sampling and costs must be considered; despite this, it was not associated with better TA [36]. Model-optimized dosing is more complex and specific software is needed. Therefore, in our opinion, a standard stratified dosing regimen with higher doses for the well-defined risk groups—such as patients with CVVH and ARC—needs to be developed and validated. Nevertheless, as long as such a stratified dosing regimen is not available, TDM might prove useful, especially in those subgroups at risk of underexposure.

## 5. Conclusions

Despite the fact that adequate median fluconazole exposure was attained in critically ill patients—even for pathogens with a relatively high MIC value of 4 mg/L—subtherapeutic exposure was observed in 13%–27% of fluconazole trough concentrations. Underexposure was mainly due to low administered doses (mg/kg), ARC, and CRRT, even for targets based on a MIC value of 2 mg/L. In our opinion, adequate doses based on bodyweight (in mg/kg) should be administered, and future research efforts should focus on the specific subgroups at risk of underexposure.

## Figures and Tables

**Table 1:** Demographics, sample information, and variability.

Patient Characteristics (*n* = 43)	
Age (years), median (IQR)	66 (58–70)
Sex (male), *n* (%)	28 (65)
Body weight (kg), median (IQR)	70 (61–87)
BMI (kg/m^2^), median (IQR)	25 (21–28)
APACHE II score (*n* = 41),	
median (IQR)	18 (14–24)
*n* (%), < 15	11 (27)
≥15	30 (73)
Length of ICU-stay (days), median (IQR)	22 (14–37)
Number of patients who received a loading dose, *n* (%)	
Yes	25 (58)
No	14 (33)
Unknown (transfer to other hospital)	4 (9)
Number of patients that received a loading dose (yes/no), *n* (%)	
Yes	18 (78)
No	5 (22)
Number of patients with at least one subtherapeutic	17 (40)
(<15 mg/L) fluconazole trough concentration, *n* (%)	
Fluconazole Concentrations (*n* = 288)	
Number of samples per patient, median (IQR)	7 (3–10)
Fluconazole C_min_ (mg/L), median (IQR)	22.9 (14.7–35.2)
Fluconazole dose (mg/kg), median (IQR)	5.3 (4.5–7.0)
Fluconazole dose (mg), median (IQR)	400 (400–400)
Fluconazole dose (mg), *n* (%)	
50	9 (3)
200	28 (10)
400	206 (72)
600	1 (0.35)
800	40 (14)
1000	1 (0.35)
1200	3 (1)
Administration route (PO), *n* (%)	28 (10)
SOFA score on day of sampling (*n* = 273), median (IQR)	7 (4–11)
Variability	
%CV intrasubject	28.3
%CV intersubject	50.5

IQR: interquartile range; ICU: intensive care unit; PO: oral; %CV: coefficient of variation; C_min_: trough concentration.

**Table 2:** Renal function and renal replacement therapy.

Renal Function During Sample Collection	Samples (*n* = 288)
eGFR (CKD-Epi) (mL/min/1.73 m^2^), median (IQR)	81 (41–101)
CrCL (Cockcroft-Gault) (mL/min), median (IQR)	83 (47–118)
Serum creatinine concentration (mg/dL), median (IQR)	0.91 (0.66–1.52)
Urea concentration (mg/dL), median (IQR)	51 (34–97)
Measured 24 h clearance (mL/min), median (IQR), *n* = 190	63 (23–101)
Number of samples while ARC (based on CKD-Epi), *n* (%)	94 (33)
Number of samples on CVVH, *n* (%) Fluconazole concentration (mg/L), median (IQR) Fluconazole dose (mg), median (IQR) Fluconazole dose (mg/kg), median (IQR)	37 (13) 14.9 (7.4–17.5) 800 (400–800) 6.7 (5.8–10.3)
Number of samples on IHD, *n* (%)	9 (3)

IQR: interquartile range; eGFR: estimated glomerular filtration rate; CrCL: creatinine clearance; ARC: augmented renal clearance; CVVH: continuous venovenous hemofiltration; IHD: intermittent hemodialysis.

**Table 3:** Significant covariates based on multivariate analyses using generalized estimating equations.

Significant Covariate	*p*-Value	Beta-Value
Multivariate analysis 1: ARC as CrCL_24h-1_ > 130 mL/min (*n* = 190)		
Dose previous administration (mg/kg)	0.010	1.30
CVVH	<0.0001	−25.96
ARC	0.002	−9.17
Multivariate analysis 2: ARC as eGFR (CKD-Epi) > 96.5 mL/min/1.73 m^2^ (*n* = 288)		
Dose previous administration (mg/kg)	<0.0001	1.26
Mode of administration	0.033	5.79
CVVH	0.006	−10.56
ARC	0.002	−2.59
Alkaline phosphatase	0.013	0.04

ARC: augmented renal clearance; CrCL: creatinine clearance; CVVH: continuous venovenous hemofiltration; eGFR: estimated glomerular filtration rate.

**Table 4:** Target nonattainment, depending on loading dose and day of fluconazole treatment.

	Loading Dose			
	Yes (*n* = 161 *)		No (*n* = 90 *)	
	Cmin < 15 mg/L	Cmin > 80 mg/L	Cmin < 15 mg/L	Cmin > 80 mg/L
Day 3	4/11 (36%)	0/11 (0%)	1/2 (50%)	0/2 (0%)
Day 4	4/12 (33%)	0/12 (0%)	2/4 (50%)	0/4 (0%)
Day 5	3/13 (23%)	0/13 (0%)	4/5 (80%)	0/5 (0%)
Day 6	4/15 (27%)	0/15 (0%)	2/7 (29%)	0/7 (0%)
Day 7	4/18 (22%)	0/18 (0%)	2/5 (40%)	0/5 (0%)
Day 8	2/17 (12%)	0/17 (0%)	2/6 (33%)	0/6 (0%)
Day 9	1/17 (6%)	1/17 (6%)	2/6 (33%)	0/6 (0%)
Day 10	0/11 (0%)	0/11 (0%)	2/6 (33%)	0/6 (0%)
Day > 10	4/47 (9%)	0/47 (0%)	12/49 (25%)	0/49 (0%)

* In 37 samples (four patients) it could not be assessed if a loading dose was administered (as patients were transferred from another hospital to ours).

## Data Availability

Individual participant data that underlie the results reported in this article are available from the corresponding author upon reasonable request, providing the request meets local ethical and research governance criteria after publication. Patient-level data will be anonymized and study documents will be redacted to protect the privacy of trial participants.
